# Editorial: Antimicrobial resistance in veterinary medicine: epidemiology, economic impact, and mitigation strategies

**DOI:** 10.3389/fvets.2026.1783072

**Published:** 2026-01-30

**Authors:** Benti Deresa Gelalcha

**Affiliations:** Department of Biomedical and Diagnostic Sciences, College of Veterinary Medicine, University of Tennessee, Knoxville, TN, United States

**Keywords:** alternative therapeutics, antimicrobial resistance, genomic surveillance, One Health, veterinary medicine

Antimicrobial resistance (AMR) is a global health challenge of the twenty-first century, undermining the effectiveness of drugs that are critical to both human and animal health (1, 2). AMR occurs when microorganisms evolve to withstand antimicrobial drugs to which they were previously susceptible (3). This process is accelerated by antimicrobial use across human, animal, and environmental sectors (4–6). Widely cited estimates project that, without effective action, AMR could cause millions of deaths and trillions in economic loss globally by mid-century (7, 8). This makes AMR a major concern for public health and sustainable development alike (9). Veterinary medicine plays a pivotal role in this landscape because animals can serve as reservoirs of resistant organisms and resistance determinants that disseminate through food systems, clinical settings, and the environment (6). The Research Topic was launched to compile new evidence and integrated approaches to AMR surveillance, impact assessment, and identify evidence-based intervention strategies across diverse veterinary contexts.

## Aims and scope of the Research Topic

The primary objective of this Research Topic was to provide a multidisciplinary platform for advancing understanding of AMR dynamics within veterinary medicine while explicitly linking microbiological, epidemiological, economic, and management perspectives. The Research Topic sought to address three interconnected pillars: (i) the epidemiology and genomic surveillance of AMR pathogens in animal populations; (ii) drivers of antimicrobial use and resistance selection; and (iii) AMR mitigation strategies. The 12 articles published in this Research Topic collectively reflect the geographic, animal species, and methodological diversity, reflecting the complexity of AMR.

## The epidemiology and genomic surveillance of AMR pathogens in animal populations

A fundamental pillar of AMR science is robust epidemiological characterization of resistance patterns and determinants. Several papers in this Research Topic contribute to that mission by applying genomic, molecular, and phenotypic approaches to delineate resistance landscapes in different animal reservoirs (food-producing animals, companion animals, and wildlife). Several contributions provide compelling evidence of persistent and emerging resistance patterns in bacterial pathogens of veterinary importance, underscoring the continued selection pressure exerted by antimicrobial use in animal systems.

Robust epidemiological surveillance remains foundational to understanding AMR emergence and spread. Capacity-building efforts, as described by Ouoba et al., demonstrated the effectiveness of the FAO ATLASS tool in strengthening national AMR surveillance systems in Sub-Saharan Africa. The study emphasizes the significance of standardized surveillance frameworks and institutional investment for data-driven decision-making and stewardship.

Several studies characterize resistance patterns in food-producing animals using molecular and phenotypic approaches. *Poultry production systems are highlighted by* Ringenier et al., who document the continued presence of fluoroquinolone-non-susceptible Escherichia coli in broilers, underscoring the persistence of clinically relevant resistance despite the absence of use of the antibiotics in that class. Similarly, Barrientos-Villegas et al. also reported in their systematic review the widespread presence of fluoroquinolone resistance in *Salmonella* from livestock (cattle, pigs, and poultry) in South America. This work synthesized genotypic and phenotypic resistance trends with implications for both animal and human health, calling for the need for ongoing surveillance, monitoring, and stronger restrictions on the use of these classes of antibiotics.

The emergence and dissemination of mobile resistance determinants (plasmid-mediated resistance genes) are further illustrated by Habib et al.. They reported the first detection and genomic characterization of *mcr-1-*mediated colistin resistance in *Salmonella Infantis* in a broiler production system in the United Arab Emirates. Similarly, environmental and wildlife reservoirs are highlighted by Zhang et al., who describe a high prevalence of plasmid-mediated fosfomycin resistance and several other resistance determinants among *Escherichia coli* isolated from waterfowl in China, showing ecological connectivity in AMR transmission.

AMR is not confined to food animal systems. Seo et al.. report antimicrobial resistance profiles of *Staphylococcus* spp. and *E. coli* from companion animals (dogs and cats) in South Korea and reported a high prevalence of resistance. The study emphasizes the importance of including small-animal veterinary practice in national surveillance frameworks to get a true picture of resistance status. Similarly, Tan et al. describe the prevalence and multidrug resistance characteristics of *Streptococcus suis* among local breeds in China. The study reported widespread occurrence of multidrug resistance, contributing to understanding endemic resistance in clinically and economically significant pathogens. These works demonstrated the importance of integrative surveillance that combines genomic characterization, phenotypic profiling, and cross-species contexts for a better understanding of the status and diversity of AMR in animal systems.

## Drivers of antimicrobial use and resistance selection

Interpreting resistance patterns requires an understanding of antimicrobial use behaviors and management practices shaping antimicrobial use. In this line, Adhikari et al.. identified complex interactions between socio-economic, knowledge, and regulatory envelopments that drive antibiotic use patterns in small- and medium-sized poultry farms in Nepal. The study illustrates the contextual complexity of antimicrobial stewardship in low-resource settings and aligns with evidence that stewardship must be sensitive to socio-economic contexts (10).

Experimental and field-based studies further link antimicrobial use to resistance selection. Circella et al. demonstrate that enrofloxacin administration under simulated field conditions can select for resistant *Pasteurella multocida* in rabbits, providing mechanistic evidence connecting use practices to resistance outcomes. In dairy systems, Abdelfattah et al. associate antimicrobial drug use with resistance in fecal commensals from dairy cows, providing evidence of selection at the herd level with implications for food safety and microbial ecology. These studies showed that resistance emergence is influenced by management and antimicrobial stewardship practices, and ecological conditions at the point of use.

## AMR mitigation strategies

*Reducing antimicrobial reliance while maintaining animal health and productivity is central to sustainable AMR mitigation. In a* clinical field trial examining wound healing after disbudding dairy calves with or without antimicrobial spray, Bijkerk et al. showed that effective wound management can be achieved without routine antimicrobial spray following calf disbudding, supporting preventive and management-based interventions. In equine medicine, Khalid et al. investigated the effect of bacteriophage cocktails to treat wound infections caused by drug-resistant bacteria in *Arabian horses* and reported a promising biologically targeted alternative to traditional antibiotics. This approach shows how molecular understanding of host–pathogen interactions can inform therapeutic innovation. These works align with broader calls for integrated mitigation frameworks, such as improved management, preventive care, and alternatives to antimicrobials within veterinary science.

**Figure 1:**
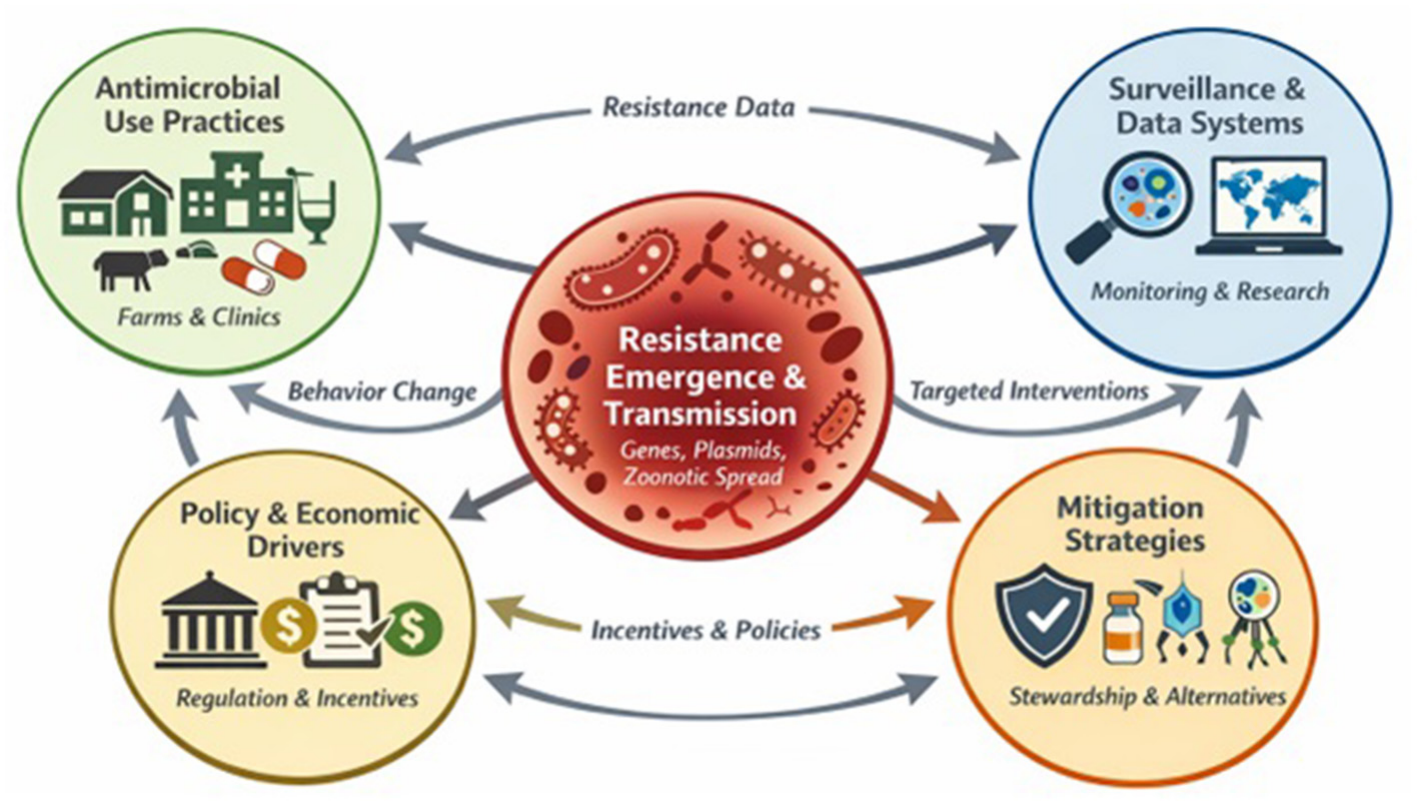
An integrative framework for understanding and addressing antimicrobial resistance in veterinary systems, as outlined in this collection of papers.

These interconnected dynamic relationships among antimicrobial use, behaviors, resistance emergence, surveillance, mitigation strategies, and policy/economic drivers in veterinary systems are summarized in Figure 1.

## Conclusion

Although the economic burden of AMR is fairly well-documented in human health, veterinary contexts have remained less explored. The studies highlighted in this editorial show implications of AMR for treatment efficiency, costs, and farm sustainability; however, none directly measure the economic burden of AMR in veterinary systems. Evidence from this Research Topic supports antimicrobial stewardship strategies, including monitoring, regulation, incentives for prudent use, and investment in surveillance, which are essential for effective AMR control. The articles in this Research Topic demonstrate that addressing AMR in veterinary medicine requires a layered approach that integrates genomic epidemiology, behavioral science, economic incentives, and policy action. They also emphasize the importance of One Health thinking by showing how resistant organisms move across species boundaries and ecological niches.
